# Chronotype and Cardiometabolic Parameters in Patients with Bipolar Disorder: Preliminary Findings

**DOI:** 10.3390/jcm12175621

**Published:** 2023-08-28

**Authors:** Andrea Aguglia, Antimo Natale, Benedetta Conio, Clio Franziska De Michiel, Alessio Lechiara, Fabrizio Pastorino, Laura Fusar-Poli, Alessandra Costanza, Andrea Amerio, Mario Amore, Gianluca Serafini

**Affiliations:** 1 Department of Neuroscience, Rehabilitation, Ophthalmology, Genetics, Maternal and Child Health, Section of Psychiatry, University of Genoa, 16132 Genoa, Italy; clio.franziska@libero.it (C.F.D.M.); lechiara.alessio@gmail.com (A.L.); fabriziopatorino.ge@gmail.com (F.P.); andrea.amerio@unige.it (A.A.); mario.amore@unige.it (M.A.); gianluca.serafini@unige.it (G.S.); 2 IRCCS Ospedale Policlinico San Martino, 16132 Genoa, Italy; benedetta.conio@hsanmartino.it; 3 Department of Psychiatry, Faculty of Medicine, Geneva University (UNIGE), 1211 Geneva, Switzerland; antimo.natale@yahoo.it (A.N.); alessandra.costanza@unige.ch (A.C.); 4 Department of Brain and Behavioral Sciences, University of Pavia, 27100 Pavia, Italy; laura.fusarpoli@unipv.it; 5 Department of Psychiatry, Adult Psychiatry Service (SPA), University Hospitals of Geneva (HUG), 1211 Geneva, Switzerland; 6 Department of Psychiatry, Faculty of Biomedical Sciences, University of Italian Switzerland (USI), 6900 Lugano, Switzerland

**Keywords:** bipolar disorder, chronotype, eveningness, metabolic parameters, cardiovascular risk

## Abstract

Cardiometabolic alterations are very common in bipolar disorder (BD). The aim of this study was to investigate the relationship between chronotype and cardiometabolic parameters in patients with a primary diagnosis of BD. This study is an observational clinical investigation including 170 subjects consecutively admitted to the Psychiatric Inpatient Unit of the IRCCS Ospedale Policlinico San Martino (Genoa, Italy), recruited over a period of 48 months. A psychometric tool assessing chronotype was administered and blood tests were performed. Furthermore, the atherogenic coefficient ((total cholesterol–HDL cholesterol)/HDL cholesterol), and Castelli risk index-I (total cholesterol/HDL cholesterol) and -II (LDL cholesterol/HDL cholesterol) were calculated. Patients with BD and an eveningness chronotype showed a higher body mass index, total and low-density lipotrotein cholesterol compared to patients with BD and an intermediate or morning chronotype. Furthermore, the Atherogenic Coefficient and Castelli Risk-Index I–II were found to be higher in bipolar patients with an evening chronotype. The role of chronotype in the development of obesity and cardiovascular risk is, therefore, a relationship worth being investigated, especially in the context of BD, to ameliorate the clinical and therapeutic approach, aiming at increasing the quality of life and reducing the mortality.

## 1. Introduction

Bipolar disorder (BD) is a highly prevalent cyclic and recurrent psychiatric disorder characterized by repetitive affective relapses, specifically major depressive and (hypo)manic episodes, during the clinical course of the disorder [1]. BD ranks as the 17th leading source of disability among all diseases worldwide and it is associated with high rates of premature mortality, due to both medical comorbidities, especially cardiometabolic clinical conditions, and high suicide rates.

The Social Zeitgeber Theory of Mood Disorders, advanced in the late 1980s, postulates that changes in the sleep–wake schedule secondary to stressful events or environmental changes (such as seasonality, longer daylight exposure or increase in temperature) are capable of altering the molecular and cellular rhythms in vulnerable individuals, causing a new onset or an affective recurrence in BD [2,3,4]. The circadian system works across multiple levels in the genome, cells and organs, leading to a homeostasis of physiological and behavioral systems. The dysregulation of circadian rhythm has been identified as a potential risk factor in the development of mood disorders, especially BD [5], being also implicated in all stages of the development of mood disorders, as described in a recent review [6]. However, the mechanisms by which circadian rhythm disruptions might lead to mood changes are still unclear, although several clock genes have been identified. It is possible that the genetic/biological vulnerability of circadian rhythms may influence mood through dysfunction in immune regulation, monoamine transmission (such as serotonin and dopamine), neurogenesis and the hypothalamic–pituitary–adrenal (HPA) axis [7,8].

Circadian rhythm and chronotype are strictly related [9,10]. Chronotype is referred to a diurnal preference, defined as individual variation in the preferred timing of the sleep–wake cycle. It reflects individual variability in the phase of entrainment and is associated with variations of a physiological nature, such as the rhythm of body temperature and hormone secretion [11]. More precisely, three (or five) chronotypes can be identified: morningness (moderate and definite morning chronotype), eveningness (moderate and definite evening chronotype) and intermediate [12]. Chronotype has recently emerged as a potential significant factor in the pathogenesis and onset of BD and, consequently, also has an impact in the prognosis, illness course (possible predictor of suicidal ideation) and therapeutic approaches [13,14,15,16]. Indeed, recent studies showed a higher prevalence of delayed sleep–wake patterns, suggesting a preference for the eveningness chronotype in patients with BD [9], related to larger daily sleep debt, a great need for sleep and morning sleepiness [17], and is considered a poor prognostic factor [18]. Furthermore, sleep disorders are recognized as the main residual symptomatology, reported also in the euthymic phase, leading to other affective symptoms, cognitive impairment, decreased quality of life and functioning, higher risk of mood recurrences and worsening of cardiovascular/metabolic parameters [19,20,21,22].

It is well known that cardiovascular and metabolic complications are reported in patients with BD, due to several factors, including pharmacological treatment with second-generation antipsychotics, shared biological, genetic and inflammatory mechanisms, unhealthy lifestyles (i.e., absence of physical activity, use of nicotine, alcohol or illicit substances, increased caloric intake, unbalance of protective nutrient-dense foods, “Western” style diet), and also multiple affective recurrences [23,24,25,26]. It is not yet clear whether this is due to a common genetic vulnerability or because of lifestyles; the most likely hypothesis is an intertwining of the two factors [27]. Furthermore, in recent studies, sleep disturbances and chronotype have been associated with the development of cardiometabolic diseases correlated to increased risk of hypertension, obesity, type 2 diabetes mellitus, insulin resistance, and the metabolic syndrome [28,29,30,31,32]. Few studies have been conducted to establish a potential relationship between circadian rhythm, chronotype, mood and cardiometabolic and endocrine functions. Recently, a relationship between circadian characteristics, sleep qualities, and metabolic parameters was demonstrated on patients with schizophrenia [33]. This clinical aspect needs more investigation, especially in patients with BD. Therefore, the aim of this study was to investigate the relationship between chronotype and cardiovascular/metabolic parameters in patients with a primary diagnosis of BD, assuming a possible significant association between metabolic and cardiovascular indexes and an eveningness chronotype.

## 2. Materials and Methods

### 2.1. Study Design

For the purpose of this cross-sectional study, all patients with a primary diagnosis of BD, consecutively admitted to the Department of Neuroscience, Rehabilitation, Ophthalmology, Genetics, Maternal and Child Health, Section of Psychiatry, Istituto di Ricovero e Cura a Carattere Scientifico Ospedale Policlinico San Martino (Genoa, Italy) from January 2019 to December 2022, were recruited.

To participate in the study, the following inclusion criteria were considered: having a primary psychiatric diagnosis of BD type I or II, according to the Diagnostic and Statistical Manual of Mental Disorders, 5th edition (DSM-5) and formulated by expert psychiatrists in an inpatient clinical setting [34], being 18 years of age or older, and providing written informed consent to participate in the study. The exclusion criteria were: (a) bipolar and related disorders secondary to psychoactive substances or physical diseases; (b) schizophrenia and related disorders; (c) pregnancy or recent childbirth, (d) any clinical condition interfering with the ability to fill out the assessment, such as the presence of cognitive deficits, causing linguistic and comprehension problems; (e) any severe neurological disorder, including recent acute neurological injury, an intellectual disability or a major neurocognitive disorder; (f) acute clinical medical conditions or having a history of cardiovascular/metabolic diseases prior to being diagnosed with BD; and (g) the refusal or inability to provide written informed consent.

All patients received detailed information about the objectives and methodology of this study, and signed a written informed consent prior the participation. The study was approved by the Local Ethical Review Board and was conducted according to the guidelines from the Declaration of Helsinki, as revised in 2013 [35].

### 2.2. Sample

One-hundred and seventy patients with BD were included in this study. Basic sociodemographic characteristics were obtained through the administration of a semi-structured interview including gender, current age, marital and occupational status, educational level and living condition.

Furthermore, at the index visit, weight (undressed and fasting) and height (barefoot) were measured, and body mass index (BMI), defined as the ratio of body weight (in kilograms) and height (in meters squared), was calculated. Lastly, blood pressure was measured. Two blood pressure measurements were obtained by using a mercury sphygmomanometer: the first was in a lying position, while the second was in a seated position, at least 2 min after the first measurement. The mean blood pressure of the two measurements was used. The attending physician performed all procedures in a hospital setting.

A blood draw for routine blood examination was performed at hospital admission for inpatients as a part of the clinical management routine. Blood samples were taken in the morning (between 7 and 8:30 am), at the time of evaluation of chronotype, from a forearm vein. Furthermore, patients were fasting for the previous 12 h, and patients who were not fasting were rescheduled for the day after. For each patient, about 3 mL of blood was collected in hemogram tubes containing EDTA. Blood exams included glucose, triglycerides, total cholesterol, low-density lipoprotein (LDL) and high-density lipoprotein (HDL) cholesterol. Blood samples were drawn in our psychiatric unit clinic and examined in the laboratory unit, Istituto di Ricovero e Cura a Carattere Scientifico Ospedale Policlinico San Martino, Genoa, Italy.

Furthermore, atherogenic coefficient ((total cholesterol–HDL cholesterol)/HDL cholesterol), and Castelli risk index-I (total cholesterol/HDL cholesterol) and -II (LDL cholesterol/HDL cholesterol) were calculated [36,37,38].

### 2.3. Psychometric Tools

Chronotype was assessed in the euthymic phase, defined as a Hamilton Depression Rating Scale total score (17-items) < 8, Young Mania Rating Scale total score < 6 and Clinical Global Impression-Bipolar Scale < 4.

The Morning Eveningness Questionnaire (MEQ)–Italian Version, a self-reported questionnaire, previously translated and validated [12,39,40], including questions about sleep habits and personal timing preferences for daily activities, was administered to all patients. The questionnaire can be used to determine whether the responder has an evening chronotype, also called late chronotype, in which they prefer to be active in the evening, and sleep and wake up late (score between 16 and 41); an intermediate chronotype (neutral or neither type), in which they have no preference for morning or evening (scores between 42 and 58); or a morning chronotype, also known as early chronotype, in which they prefer to be active in the morning and sleep and wake early (scores between 59 and 86). So, morning chronotype individuals achieve peak physical and mental performance in the early part of the day after waking up while evening chronotype individuals have the best mental and physical performance before sleeping [41].

### 2.4. Statistical Analysis

All of the statistical analyses were performed using the Statistical Package for Social Sciences (IBM Corp., Armonk, NY, USA) for Windows 25.0 with the significance fixed at *p* < 0.05 (two-tailed).

All characteristics investigated were represented as absolute number and percentage for categorical variables, while mean and standard deviation (SD) were used for continuous variables. The Kolmogorov–Smirnov test was used to evaluate where parametric and non-parametric test was more appropriate for our purpose.

Therefore, the sample was divided into three subgroups based on the chronotype preference to examine potential differences in terms of sociodemographic and cardiovascular/metabolic parameters. In order to analyze the differences between these three subgroups, ANOVA with the Bonferroni post-hoc test for pairwise comparisons was preferred for continuous variables. Finally, a logistic regression with evening chronotype as dependent variable was performed to evaluate whether metabolic or cardiovascular parameters were statistically associated.

The probability of entering the equation was set at 0.05.

## 3. Results

Our study population consisted of 170 patients with a primary diagnosis of BD, of which eighty-nine (N = 89, 52.4%) were males with a current mean age of 51.96 years (±14.59). Furthermore, twenty-two (N = 22, 12.9%) reported an eveningness chronotype, seventy-six (N = 76, 44.7%) had an intermediate chronotype and seventy-two (N = 72, 42.4%) showed a morningness chronotype.

Regarding the comparison of sociodemographic characteristics, only the current age was found to be statistically significant in relation to chronotype; more precisely, patients with BD and an evening chronotype were younger compared to the other two subgroups (42.18 ± 14.40 vs. 52.89 ± 14.21 vs. 53.97 ± 14.03, *p* = 0.003). No other statistical differences were found. All sociodemographic characteristics of the whole sample and chronotype differences were summarized in Table 1.

Regarding metabolic and cardiovascular parameters, several statistically significant differences were found. First, patients with BD and an evening chronotype showed a higher BMI compared to patients with BD and an intermediate or morning chronotype (26.69 ± 6.05 vs. 23.43 ± 3.71 vs. 24.46 ± 4.30, *p* = 0.008). Second, total cholesterol (205.18 ± 45.21 vs. 168.82 ± 35.69 vs. 170.43 ± 42.64), *p* = 0.001) and LDL-cholesterol (129.68 ± 39.45 vs. 100.36 ± 27.82 vs. 101.53 ± 34.29, *p* = 0.001) were significantly found to be higher in patients with BD and an evening chronotype, compared to the other two subgroups. Furthermore, the Atherogenic Coefficient, an index given by the ratio of non-HDL to HDL cholesterol, was calculated as significantly higher in bipolar patients with an evening chronotype (3.59 ± 1.32 vs. 2.70 ± 1.22 vs. 2.51 ± 1.27, *p* = 0.002). Finally, Castelli Risk-Index-I and -II, used to assess CV risk, were found to be significantly higher in bipolar patients with an evening chronotype (4.59 ± 1.32 vs. 3.70 ± 1.22 vs. 3.51 ± 1.27, *p* = 0.002 and 2.92 ± 1.10 vs. 2.24 ± 0.96 vs. 2.12 ± 0.93, *p* = 0.003, respectively). All comparisons are summarized in Table 2.

A logistic regression analysis with eveningness chronotype as the dependent variable generated the following ORs, corrected for age and gender: current age, OR = 0.939, 95% CI (0.902–0.976), *p* = 0.002; body mass index, OR = 1.107, 95% CI (1.005–1.220), *p* = 0.038; and LDL-c, OR = 1.023, 95% CI (1.008–1.039), *p* = 0.003. The R^2^ Nagelkerke was 0.388 while the X^2^ Hosmer–Lemeshow test was 11.272.

## 4. Discussion

The aim of our study was to evaluate the possible interactions between chronotype, metabolic parameters and cardiovascular risk in a sample of patients with a primary diagnosis of BD. These preliminary findings could help clinicians and researchers to clarify the potential relationship between BD and cardiometabolic comorbidities, identifying a subgroup of patients with BD more prone to develop these alterations. In patients suffering from BD and having an eveningness chronotype, alternative therapeutic approaches, both pharmacological and non-pharmacological, could help to delay the occurrence of these complications. For example, the use of third-generation antipsychotics or antidepressants with less impact on weight gain and metabolic parameters, or a psychoeducation more focused on the unhealthy lifestyle or sleep hygiene could provide a better management of long-term treatment of these subgroups of patients with BD.

The findings of our study have found that the eveningness chronotype in patients with BD is associated with increased BMI, total cholesterol and LDL-c values, as well as the cardiovascular indexes investigated. This potential relationship in the context of BD is poorly investigated in the literature, and our data are in line with the few studies conducted so far. Indeed, to our knowledge, only two recent studies on chronotype in patients with BD showed a significant association between eveningness chronotype and a higher prevalence of total body fat and obesity [18,42]. On the contrary, a recent systematic review summarized studies investigating the relationship between chronotype and obesity on the general population. Findings confirmed that subjects with eveningness chronotype had an increased BMI and weight [43], suggesting a potential role for chronotype on peripheral mechanisms of metabolic homeostasis.

In this context, our findings could be interpreted on the basis of both behavioral and biological mechanisms. On one hand, it is well known that the eveningness chronotype is associated with unhealthy lifestyle habits [44,45]; several studies on healthy subjects reported that the presence of an eveningness chronotype is related to a less active behavior and the consumption of sweet and fatty foods, while subjects with a morningness chronotype are more inclined to practice physical activity, have a longer sleep and eat more fruits and vegetables [44,46,47]. Other studies reported that subjects with an eveningness chronotype normally consume meals later, usually in front of the TV, eating longer and often skipping meals, and with an increased glycemic peak [48,49]. It is possible to extend these data also to the patients affected by BD, a psychopathological condition which is already known to have an unhealthier lifestyle than the general population [50]. Further comparative studies are needed to clarify the possible role of lifestyles in determining the tendency of patients with BD and an eveningness chronotype to have significant metabolic alterations, weight gain and increased cardiovascular risk.

Moving to biological mechanisms, the circadian rhythm allows the body to prepare for environmental changes due to the phases of the day by anticipating the various physiological, cellular and behavioral processes [51,52]. The suprachiasmatic nucleus (SCN) serves as the master circadian pacemaker, synchronizing peripheral clocks through neural (including other clocks placed in the brain, such as the amygdala and thalamus) and endocrine signaling mechanisms (peripheral oscillators identified as endogenous oscillators), and aligning daily rhythms in physiology and behavior to day/night or sleep/wake cycles, shift work, jet lag or social life identified as influencing exogeneous factors [51,52,53]. It converts light signals to rhythmic output, which drives rhythms in the autonomic nervous system as well as cortisol and melatonin levels, so as to keep normal circadian periods. Melatonin production is suppressed by light and, therefore, its concentrations increase mainly at night, with a peak in the middle of the night. Melatonin, the major regulator of the sleep/wake cycle, plays important physiological and pharmacological roles in the control of neuronal plasticity and neuroprotection, influencing several vital functions, such as body temperature, metabolic and glucose homeostasis, and immunological and anti-inflammatory functions [54]. So, it is very important to remember that the long-lasting reduction in melatonin secretion is often associated not only with disturbances in the sleep/wake rhythm, but also with a significant weight gain [55].

Recently, specific differences in melatonin rhythms in individuals of different chronotypes were reported; an eveningness chronotype has later melatonin rhythms with decreased mean levels and peaks [56,57]. This is probably caused by the large differences in daily light exposure between chronotypes, i.e., the morningness chronotype has more minutes of daily bright light exposure (>1000 lux) than the eveningness chronotype [58]. Lastly, chronotype was identified as the strongest predictor of sleep disturbances, being a worsening of sleep quality and severe insomnia, mostly related to subjects with evening chronotype [59].

Cortisol, produced by the activation of the hypothalamic–pituitary–adrenal (HPA) axis, has its peak concentration upon awakening and its nadir during the night. It influences all peripheral processes and is especially activated in stressful situations [60]. Therefore, due to the bidirectional communication between serotonin and circadian rhythm and the influence of stress-induced changes in cortisol, the circadian rhythm has a significant role in metabolism regulation and process, including glucose and lipid homeostasis, insulin sensitivity, and energy expenditure [61,62,63]. Vulnerable clock genes and genetic variants have been identified in the literature [64,65].

Several studies have demonstrated a link between chronotype and functionality of the central clock. In fact, it has been reported that the eveningness chronotype is associated with a degree of misalignment between the internal clock and the external environmental conditions, which leads to important interferences between central and peripheral rhythms [66,67,68]. The consequences of this misalignment would lead to an increased risk of developing metabolic diseases (such as diabetes, obesity and the metabolic syndrome) through an imbalance in cortisol concentrations and increased inflammation [29,69,70,71].

Indeed, chronotype influences the release of glucocorticoids from the adrenal gland. Several studies have underlined that chronotype affects the diurnal profile of cortisol regulated by the HPA axis. For example, morningness chronotype hashigher awakening cortisol peaks and a significantly greater cortisol response to stress than eveningness types [72,73]. It should not be forgotten that glucocorticoids are important regulators of lipid and glucose metabolism and that alterations in the function of the HPA axis predispose to obesity [74]. Abnormalities of the HPA axis are associated with insulin resistance and hyperglycemia, which could lead to endocrine and metabolic deseases [75]. It is important to underline that the associations between chronotype and HPA axis function are still understudied. Some authors suggest that differences in stress-induced neuroendocrine reactivity patterns may underlie the association between chronotype and metabolic functions, and may contribute to the development of metabolic derangements [29]. A longitudinal study suggested cortisol and gene circadian rhythm as diagnostic biomarkers and therapeutic targets for BD [31].

Additionally, several studies indicate strong links between circadian rhythms and the immune system. For example, peripheral clocks have been shown to be present in several hematopoietic cell lineages including macrophages and lymphocytes, determining the response to pathogens and their movement in and out of the bloodstream [76]. Immune parameters are characterized by daily variations, demonstrating a circadian pattern [9]. Immune cell activity is under circadian control, with the mediation of sympathetic nerve fibers and stress hormones like glucocorticoids [29]. Recent studies have highlighted an increase in c-reactive protein (c-RP) levels in subjects with an eveningness chronotype, also in the context of BD, demonstrating an association between elevated inflammatory levels and eveningness chronotype [29,77]. Circadian rhythm disturbance is related to an increased lipid peroxidation in BD, due to oxidative stress [78]. Furthermore, higher c-RP concentrations, increased triglycerides levels and higher total body fat mass are significantly associated with eveningness chronotype compared to morningness and intermediate types [70]. Therefore, it is possible to think that inflammation may also contribute to the development of obesity in subjects with an eveningness chronotype, also given the well-known association between low-grade chronic inflammation and the development of metabolic diseases [79].

Despite the clinical relevance of our findings, several limitations should be discussed. First, our study has a cross-sectional design, therefore, it is not possible to draw any inference on the temporal or causal relationship between the variables considered. Second, some important parameters that may modify the metabolic profile (i.e., substance use, psychiatric comorbidities, unhealthy lifestyles) were not included in the analyses due to several missing values. Third, a power analysis has been not conducted for this study; therefore, the sample size might have been not sufficiently large to detect significant differences. Finally, our data are limited to those derived from a single research center and inpatient unit; therefore, the findings should not be generalized, also due to the absence of outpatients or comparison with healthy controls.

## 5. Conclusions

The present study lays the ground for a novel line of research based on the evaluation of chronotype in patients affected by BD. It may find potential applications in daily clinical practice and provide numerous cues for future research. The role of chronotype in the development of obesity and cardiovascular risk is, therefore, a relationship worth being investigated, especially in the context of BD. Clinicians should take into consideration the chronotype primarily to promote healthier lifestyles in patients with eveningness chronotype; such as, for example, prescribing diets not only based on caloric intake but also on the chronotype [80]. Longitudinal investigations of the interaction between cardiometabolic parameters and chronotype in patients affected by BD may further help to clarify the potential mechanisms underlying this complex relationship, leading to the discovery of more solid links. Furthermore, it could be useful to investigate the molecular links between chronotype, inflammation, and the HPA axis, with the aim of improving personalized pharmacological and non-pharmacological interventions. Reducing the risk of obesity and cardiovascular diseases present a major challenge for clinicians and a fundamental intervention to increase the quality of life, extend the psychophysical well-being and reduce the mortality of patients with BD.

## Figures and Tables

**Table 1:** Sociodemographic characteristics of total sample and comparison among the three subgroups.

		Chronotype				
N (%) or Mean ± SD	Total Sample (N = 170)	Eveningness (N = 22)	Intermediate (N = 76)	Morningness (N = 72)	*F*	*p*
Gender (male)	89 (52.4)	12 (54.5)	43 (56.6)	34 (47.2)	1.346	0.510
Current Age (years)	51.96 ± 14.59	42.18 ± 14.40	52.89 ± 14.21	53.97 ± 14.03	6.137	0.003 *
Marital Status					11.647	0.070
Single	64 (37.6)	14 (63.6)	28 (36.8)	22 (30.6)		
Married	54 (31.8)	6 (27.3)	27 (35.5)	21 (29.2)		
Separated/Divorced	39 (22.9)	2 (9.1)	15 (19.7)	22 (30.6)		
Widowed	13 (7.6)	0 (0.0)	6 (7.9)	7 (9.7)		
Educational level (years)	11.16 ± 3.65	10.95 ± 3.33	11.84 ± 3.58	10.50 ± 3.73	2.585	0.078
Occupational status	65 (38.2)	9 (40.9)	29 (38.2)	27 (37.5)	0.083	0.959
Living with, N (%)					5.593	0.232
Alone	43 (25.2)	3 (13.6)	23 (30.3)	17 (23.6)		
Family	123 (72.4)	18 (81.8)	53 (69.7)	52 (72.2)		
Residence	4 (2.4)	1 (4.6)	0 (0.0)	3 (4.2)		

* Post-hoc Bonferroni: E > I = M.

**Table 2:** Comparison of metabolic parameters among the three subgroups in patients with bipolar disorder.

	Chronotype					
	Eveningness (N = 22)	Intermediate (N = 76)	Morningness (N = 72)	*F*	*p*	
Weight	76.84 ± 17.36	68.32 ± 14.35	69.36 ± 15.94	2.667	0.072	
Height	169.55 ± 7.38	170.16 ± 9.07	167.65 ± 9.05	1.521	0.222	
Body Mass Index	26.69 ± 6.05	23.43 ± 3.71	24.46 ± 4.30	4.966	0.008	E > I
Blood pressure						
Systolic	126.14 ± 10.23	127.64 ± 16.58	126.10 ± 13.78	0.229	0.796	
Diastolic	77.05 ± 9.34	77.32 ± 9.45	75.68 ± 9.96	0.558	0.573	
Total cholesterol	205.18 ± 45.21	168.82 ± 35.69	170.43 ± 42.64	7.597	0.001	E > I = M
HDL-c	47.23 ± 13.05	48.22 ± 11.86	50.64 ± 14.70	0.872	0.420	
LDL-c	129.68 ± 39.45	100.36 ± 27.82	101.53 ± 34.29	7.612	0.001	E > I = M
Triglycerides	123.18 ± 58.97	120.75 ± 67.10	135.46 ± 67.68	0.956	0.386	
Glucose	94.64 ± 15.59	102.39 ± 36.29	34.22 ± 22.91	1.623	0.200	
Atherogenic Coefficient	3.59 ± 1.32	2.70 ± 1.22	2.51 ± 1.27	6.336	0.002	E > I = M
Castelli Risk Index-I	4.59 ± 1.32	3.70 ± 1.22	3.51 ± 1.27	6.336	0.002	E > I = M
Castelli Risk Index-II	2.92 ± 1.10	2.24 ± 0.96	2.12 ± 0.93	5.883	0.003	E > I = M

HDL-c: High density lipoprotein cholesterol; LDL-c: Low density lipoprotein cholesterol.

## Data Availability

The data presented in this study are available on request from the corresponding author. The data are not publicly available due to privacy/ethical restrictions.

## References

[1] American Psychiatric Association (2022). Diagnostic and Statistical Manual of Mental Disorders-Text Revision: DSM-5-TR.

[2] Ehlers C.L., Frank E., Kupfer D.J. (1988). Social zeitgebers and biological rhythms. A unified approach to understanding the etiology of depression. Arch. Gen Psychiatry.

[3] Aguglia A., Borsotti A., Maina G. (2018). Bipolar disorders: Is there an influence of seasonality or photoperiod?. Braz. J. Psychiatry.

[4] Aguglia A., Serafini G., Escelsior A., Canepa G., Amore M., Maina G. (2019). Maximum Temperature and Solar Radiation as Predictors of Bipolar Patient Admission in an Emergency Psychiatric Ward. Int. J. Environ. Res. Public Health.

[5] Neves A.R., Albuquerque T., Quintela T., Costa D. (2022). Circadian rhythm and disease: Relationship, new insights, and future perspectives. J. Cell Physiol..

[6] Crouse J.J., Carpenter J.S., Song Y.J.C., Hockey S.J., Naismith S.L., Grunstein R.R., Scott E.M., Merikangas K.R., Scott J., Hickie I.B. (2021). Circadian rhythm sleep-wake disturbances and depression in young people: Implications for prevention and early intervention. Lancet Psychiatry.

[7] McClung C.A. (2013). How might circadian rhythms control mood? Let me count the ways. Biol. Psychiatry.

[8] McCarthy M.J., Gottlieb J.F., Gonzalez R., McClung C.A., Alloy L.B., Cain S., Dulcis D., Etain B., Frey B.N., Garbazza C. (2022). Neurobiological and behavioral mechanisms of circadian rhythm disruption in bipolar disorder: A critical multi-disciplinary literature review and agenda for future research from the ISBD task force on chronobiology. Bipolar Disord..

[9] Melo M.C.A., Abreu R.L.C., Linhares Neto V.B., de Bruin P.F.C., de Bruin V.M.S. (2017). Chronotype and circadian rhythm in bipolar disorder: A systematic review. Sleep Med. Rev..

[10] Takaesu Y. (2018). Circadian rhythm in bipolar disorder: A review of the literature. Psychiatry Clin. Neurosci..

[11] Zou H., Zhou H., Yan R., Yao Z., Lu Q. (2022). Chronotype, circadian rhythm, and psychiatric disorders: Recent evidence and potential mechanisms. Front. Neurosci..

[12] Horne J.A., Ostberg O. (1976). A self-assessment questionnaire to determine morningness-eveningness in human circadian rhythms. Int. J. Chronobiol..

[13] Soreca I. (2014). Circadian rhythms and sleep in bipolar disorder: Implications for pathophysiology and treatment. Curr. Opin. Psychiatry.

[14] Stange J.P., Kleiman E.M., Sylvia L.G., Magalhães P.V., Berk M., Nierenberg A.A., Deckersbach T. (2016). Specific mood symptoms confer risk for subsequent suicidal ideation in bipolar disorder with and without suicide attempt history: Multi-wave data from STEP-BD. Depress. Anxiety.

[15] Ahmad A., Anderson K.N., Watson S. (2021). Sleep and Circadian Rhythm Disorder in Bipolar Affective Disorder. Curr. Top. Behav. Neurosci..

[16] Bisdounis L., Saunders K.E.A., Farley H.J., Lee C.K., McGowan N.M., Espie C.A., Kyle S.D. (2022). Psychological and behavioural interventions in bipolar disorder that target sleep and circadian rhythms: A systematic review of randomised controlled trials. Neurosci. Biobehav. Rev..

[17] Taillard J., Philip P., Chastang J.F., Bioulac B. (2004). Validation of Horne and Ostberg morningness-eveningness questionnaire in a middle-aged population of French workers. J. Biol. Rhythm..

[18] Melo M.C., Garcia R.F., de Araùjo C.F., Luz J.H., de Bruin P.F., de Bruin V.M. (2020). Chronotype in bipolar disorder: An 18-month prospective study. Braz. J. Psychiatry.

[19] Harvey A.G. (2008). Sleep and circadian rhythms in bipolar disorder: Seeking synchrony, harmony, and regulation. Am. J. Psychiatry.

[20] Cretu J.B., Culver J.L., Goffin K.C., Shah S., Ketter T.A. (2016). Sleep, residual mood symptoms, and time to relapse in recovered patients with bipolar disorder. J. Affect Disord.

[21] Brochard H., Godin O., Geoffroy P.A., Yeim S., Boudebesse C., Benizri C., Benard V., Maruani J., Leboyer M., Bellivier F. (2018). Metabolic syndrome and actigraphy measures of sleep and circadian rhythms in bipolar disorders during remission. Acta Psychiatr. Scand..

[22] Godin O., Henry C., Leboyer M., Azorin J.M., Aubin V., Bellivier F., Polosan M., Courtet P., Gard S., Kahn J.P. (2017). Sleep quality, chronotype and metabolic syndrome components in bipolar disorders during the remission period: Results from the FACE-BD cohort. Chronobiol. Int..

[23] McIntyre R.S., Calabrese J.R. (2019). Bipolar depression: The clinical characteristics and unmet needs of a complex disorder. Curr. Med. Res. Opin..

[24] Fusar-Poli L., Amerio A., Cimpoesu P., Natale A., Salvi V., Zappa G., Serafini G., Amore M., Aguglia E., Aguglia A. (2020). Lipid and Glycemic Profiles in Patients with Bipolar Disorder: Cholesterol Levels Are Reduced in Mania. Medicina.

[25] Salvi V., Aguglia A., Barone-Adesi F., Bianchi D., Donfrancesco C., Dragogna F., Palmieri L., Serafini G., Amore M., Mencacci C. (2020). Cardiovascular risk in patients with severe mental illness in Italy. Eur. Psychiatry.

[26] Aguglia A., Salvi V., Amerio A., Gari M., Dragogna F., Mencacci C., Volpe U., Serafini G., Amore M. (2022). Number of episodes and duration of illness associated with hypertension and 10-year cardiovascular risk in patients with bipolar disorder type I. Psychiatry Res..

[27] Miola A., De Filippis E., Veldic M., Ho A.M., Winham S.J., Mendoza M., Romo-Nava F., Nunez N.A., Gardea Resendez M., Prieto M.L. (2022). The genetics of bipolar disorder with obesity and type 2 diabetes. J. Affect. Disord..

[28] McMahon D.M., Burch J.B., Youngstedt S.D., Wirth M.D., Hardin J.W., Hurley T.G., Blair S.N., Hand G.A., Shook R.P., Drenowatz C. (2019). Relationships between chronotype, social jetlag, sleep, obesity and blood pressure in healthy young adults. Chronobiol. Int..

[29] de Punder K., Heim C., Entringer S. (2019). Association between chronotype and body mass index: The role of C-reactive protein and the cortisol response to stress. Psychoneuroendocrinology.

[30] Bhar D., Bagepally B.S., Rakesh B. (2022). Association between chronotype and cardio-vascular disease risk factors: A systematic review and meta-analysis. Clin. Epidemiol. Glob. Health.

[31] Ekiz Erim S., Sert H. (2023). The relationship between chronotype and obesity: A systematic review. Chronobiol. Int..

[32] Yan X., Xu P., Sun X. (2023). Circadian rhythm disruptions: A possible link of bipolar disorder and endocrine comorbidities. Front. Psychiatry.

[33] Balcioglu S.S.K., Balcioglu Y.H., Devrim Balaban O. (2022). The association between chronotype and sleep quality, and cardiometabolic markers in patients with schizophrenia. Chronobiol. Int..

[34] American Psychiatric Association (2013). Diagnostic and Statistical Manual of Mental Disorders: DSM-5.

[35] World Medical Association (2013). World Medical Association Declaration of Helsinki: Ethical principles for medical research involving human subjects. JAMA.

[36] Brehm A., Pfeiler G., Pacini G., Vierhapper H., Roden M. (2004). Relationship between serum lipoprotein ratios and insulin resistance in obesity. Clin. Chem..

[37] Millán J., Pintó X., Muñoz A., Zuniga M., Rubiès-Prat J., Pallardo L.F., Masana L., Mangas A., Hernandez-Mijares A., Gonzalez-Santo P. (2009). Lipoprotein ratios: Physiological significance and clinical usefulness in cardiovascular prevention. Vasc. Health Risk Manag..

[38] Shilpa B., Pharm I.J., Sci B., Bhardwaj S., Bhattacharjee J., Bhatnagar M.K., Tyagi S. (2013). Atherogenic index of plasma, castelli risk index and atherogenic coefficient-new parameters in assessing cardiovascular risk. Int. J. Pharm. Biol. Sci..

[39] Cavallera G.M., Boari G. (2015). Validation of the Italian Version of the Morningness-Eveningness Questionnaire for Adolescents by A. Lancry and Th. Arbault. Med. Sci. Monit..

[40] Viticchi G., Falsetti L., Paolucci M., Altamura C., Buratti L., Salvemini S., Brunelli N., Bartolini M., Vernieri F., Silvestrini M. (2019). Influence of chronotype on migraine characteristics. Neurol. Sci..

[41] Song J., Feng P., Zhao X., Xu W., Xiao L., Zhou J., Zheng Y. (2018). Chronotype regulates the neural basis of response inhibition during the daytime. Chronobiol. Int..

[42] Soreca I., Fagiolini A., Frank E., Goodpaster B.H., Kupfer D.J. (2009). Chronotype and body composition in bipolar disorder. Chronobiol. Int..

[43] Teixeira G.P., Guimarães K.C., Soares A.G.N.S., Marqueze E.C., Moreno C.R.C., Mota M.C., Crispim C.A. (2022). Role of chronotype in dietary intake, meal timing, and obesity: A systematic review. Nutr. Rev..

[44] Patterson F., Malone S.K., Lozano A., Grandner M.A., Hanlon A.L. (2016). Smoking, Screen-Based Sedentary Behavior, and Diet Associated with Habitual Sleep Duration and Chronotype: Data from the UK Biobank. Ann. Behav. Med..

[45] Song X., Wang H., Zhang M., Meng J., Su C., Zou Q., Zhang B. (2023). Bidirectional mediation of evening energy proportion and chronotype on their associations with blood lipid levels in a Chinese population. Chronobiol. Int..

[46] Maukonen M., Kanerva N., Partonen T., Kronholm E., Konttinen H., Wennman H., Männistö S. (2016). The associations between chronotype, a healthy diet and obesity. Chronobiol. Int..

[47] Mazri F.H., Manaf Z.A., Shahar S., Mat Ludin A.F. (2019). The Association between Chronotype and Dietary Pattern among Adults: A Scoping Review. Int. J. Environ. Res. Public Health.

[48] Sato-Mito N., Sasaki S., Murakami K., Okubo H., Takahashi Y., Shibata S., Yamada K., Sato K. (2011). The midpoint of sleep is associated with dietary intake and dietary behavior among young Japanese women. Sleep Med..

[49] Reutrakul S., Hood M.M., Crowley S.J., Morgan M.K., Teodori M., Knutson K.L. (2014). The relationship between breakfast skipping, chronotype, and glycemic control in type 2 diabetes. Chronobiol. Int..

[50] Bauer I.E., Gálvez J.F., Hamilton J.E., Balanzá-Martínez V., Zunta-Soares G.B., Soares J.C., Meyer T.D. (2016). Lifestyle interventions targeting dietary habits and exercise in bipolar disorder: A systematic review. J. Psychiatr. Res..

[51] Dibner C., Schibler U., Albrecht U. (2010). The mammalian circadian timing system: Organization and coordination of central and peripheral clocks. Annu. Rev. Physiol..

[52] Patke A., Young M., Axelrod S. (2020). Molecular mechanisms and physiological importance of circadian rhythms. Nat. Rev. Mol. Cell Biol..

[53] Abreu T., Braganca M. (2015). The bipolarity of light and dark: A review on Bipolar Disorder and circadian cycles. J. Affect. Disord..

[54] Zisapel N. (2018). New perspectives on the role of melatonin in human sleep, circadian rhythms and their regulation. Br. J. Pharmacol..

[55] Biggio G., Biggio F., Talani G., Mostallino M.C., Aguglia A., Aguglia E., Palagini L. (2021). Melatonin: From neurobiology to treatment. Brain Sci..

[56] Razavi P., Devore E.E., Bajaj A., Lockley S.W., Figueiro M.G., Ricchiuti V., Gauderman W.J., Hankinson S.E., Willett W.C., Schernhammer E.S. (2019). Shift work, chronotype, and melatonin rhythm in nurses. Cancer Epidemiol. Biomark. Prev..

[57] McHill A.W., Sano A., Hilditch C.J., Barger L.K., Czeisler C.A., Picard R., Klerman E.B. (2021). Robust stability of melatonin circadian phase, sleep metrics, and chronotype across months in young adults living in real-world settings. J. Pineal. Res..

[58] Goulet G., Mongrain V., Desrosiers C., Paquet J., Dumont M. (2007). Daily light exposure in morning-type and evening-type individuals. J. Biol. Rhythm.

[59] Salfi F., D’Atri A., Tempesta D., Ferrara M. (2021). Sleeping under the waves: A longitudinal study across the contagion peaks of the COVID-19 pandemic in Italy. J. Sleep Res..

[60] Chung S., Son G.H., Kim K. (2011). Circadian rhythm of adrenal glucocorticoid: Its regulation and clinical implications. Biochim. Biophys. Acta.

[61] Barandas R., Landgraf D., McCarthy M., Welsh D. (2015). Circadian clocks as modulators of metabolic comorbidity in psychiatric disorders. Curr. Psychiatry Rep..

[62] Panda S. (2016). Circadian physiology of metabolism. Science.

[63] Poggiogalle E., Jamshed H., Peterson C. (2018). Circadian regulation of glucose, lipid, and energy metabolism in humans. Metabolism.

[64] Fishbein A., Knutson K., Zee P. (2021). Circadian disruption and human health. J. Clin. Investig..

[65] Gentry N.W., Ashbrook L.H., Fu Y.H., Ptacek L.J. (2021). Human circadian variations. J. Clin. Investig..

[66] Wittmann M., Dinich J., Merrow M., Roenneberg T. (2006). Social jetlag: Misalignment of biological and social time. Chronobiol. Int..

[67] Huang W., Ramsey K.M., Marcheva B., Bass J. (2011). Circadian rhythms, sleep, and metabolism. J. Clin. Investig..

[68] Roenneberg T., Merrow M. (2016). The circadian clock and human health. Curr. Biol..

[69] Arora T., Taheri S. (2015). Associations among late chronotype, body mass index and dietary behaviors in young adolescents. Int. J. Obes..

[70] Yu J.H., Yun C.H., Ahn J.H., Suh S., Cho H.J., Lee S.K., Yoo H.J., Seo J.A., Kim S.G., Choi K.M. (2015). Evening chronotype is associated with metabolic disorders and body composition in middle-aged adults. J. Clin. Endocrinol. Metab..

[71] Sun X., Gustat J., Bertisch S.M., Redline S., Bazzano L. (2020). The association between sleep chronotype and obesity among black and white participants of the Bogalusa Heart Study. Chronobiol. Int..

[72] Randler C., Schaal S. (2010). Morningness-eveningness, habitual sleep-wake variables and cortisol level. Biol. Psychol..

[73] Marvel-Coen J., Nickels N., Maestripieri D. (2018). The relationship between morningness–eveningness, psychosocial variables, and cortisol reactivity to stress from a life history perspective. Evolut. Behav. Sci..

[74] Hewagalamulage S.D., Lee T.K., Clarke I.J., Henry B.A. (2016). Stress, cortisol, and obesity: A role for cortisol responsiveness in identifying individuals prone to obesity. Domest. Anim. Endocrinol..

[75] Calkin C.V., Gardner D.M., Ransom T., Alda M. (2013). The relationship between bipolar disorder and type 2 diabetes: More than just co-morbid disorders. Ann. Med..

[76] Scheiermann C., Kunisaki Y., Frenette P.S. (2013). Circadian control of the immune system. Nat. Rev. Immunol..

[77] Orsolini L., Ricci L., Pompili S., Cicolini A., Volpe U. (2023). Eveningness chronotype and depressive affective emperament associated with higher high-sensitivity C-reactive protein in unipolar and bipolar depression. J. Affect. Disord..

[78] Cudney L.E., Sassi R.B., Behr G.A., Streiner D.L., Minuzzi L., Moreira J.C., Frey B.N. (2014). Alterations in circadian rhythms are associated with increased lipid peroxidation in females with bipolar disorder. Int. J. Neuropsychopharmacol.

[79] Kawai T., Autieri M.V., Scalia R. (2021). Adipose tissue inflammation and metabolic dysfunction in obesity. Am. J. Physiol. Cell Physiol..

[80] Muñoz J.S.G., Cañavate R., Hernández C.M., Cara-Salmerón V., Morante J.J.H. (2017). The association among chronotype, timing of food intake and food preferences depends on body mass status. Eur. J. Clin. Nutr..

